# Potential Cause of Decrease in Bloom Events of the Harmful Dinoflagellate *Cochlodinium polykrikoides* in Southern Korean Coastal Waters in 2016

**DOI:** 10.3390/toxins12060390

**Published:** 2020-06-12

**Authors:** Seung Ho Baek, Yunji Kim, Minji Lee, Chi-Yong Ahn, Kyung Hwa Cho, Bum Soo Park

**Affiliations:** 1 Risk Assessment Research Center, Korea Institute of Ocean Science & Technology, Geoje 53201, Korea; baeksh@kiost.ac.kr (S.H.B.); helena4818@kiost.ac.kr (Y.K.); mjlee@kiost.ac.kr (M.L.); 2 Korean Seas Geosystem Research Unit, Korea Institute of Ocean Science & Technology, Busan 49111, Korea; 3 Cell Factory Research Center, Korea Research Institute of Bioscience & Biotechnology, 125 Gwahak-ro, Yuseong-gu, Daejeon 34141, Korea; cyahn@kribb.re.kr; 4 School of Urban and Environmental Engineering, Ulsan National Institute of Science and Technology, Ulsan 44919, Korea; khcho@unist.ac.kr; 5 Marine Ecosystem Research Center, Korea Institute of Ocean Science and Technology, Busan 49111, Korea

**Keywords:** *Cochlodinium polykrikoides*, massive blooms, physico-chemical factors, Changjiang River discharge, Tongyeong coast (South Korea)

## Abstract

Blooms of the ichthyotoxic dinoflagellate *Cochlodinium polykrikoides* are responsible for massive fish mortality events in Korean coastal waters (KCW). They have been consistently present in southern KCW over the last two decades, but they were not observed in 2016, unlike in the previous years. Despite extensive studies, the cause of this absence of this dinoflagellate bloom remains largely unknown. Thus, we compared physico-chemical and biological data from along the Tongyeong coast between 2016 and the previous four years (2012–2015). The averages of water temperature and salinity in August, 2016 were significantly (*p* < 0.001) different from those in the previous years. The amount of Changjiang River discharge, which can affect the environmental conditions in the southern Korean coastal area via ocean currents, was larger than in the previous years, resulting in a reduction in the salinity level in August when blooms of *C. polykrikoides* usually occurred. Moreover, compared to previous years, in 2016, there was a weak expansion of *C. polykrikoides* blooms in the Goheung-Oenarodo area where *C. polykrikoides* blooms were annually initiated in KCW. Lastly, the strong winds from the typhoon Lionrock may also have contributed to the early termination of this dinoflagellate bloom. Together with these findings, the combination of these environmental conditions in 2016, unlike in previous years, may have inhibited the formation of *C. polykrikoides* blooms along the Tongyeong coast.

## 1. Introduction

Harmful algal blooms (HABs) are a growing environmental problem globally and have serious impacts on public health, aquatic organisms, and aquaculture industries [1,2,3]. The ichthyotoxic unarmored dinoflagellate *Cochlodinium polykrikoides* is one of the most commonly occurring HABs in coastal waters all over the world, such as in North America, the Middle East, East Asia, etc. [4,5]. This dinoflagellate can kill fishes in aquaculture tanks within 2 h by clogging fish gills when the cell abundance exceeds approximately 1000 cells mL^−1^ [6,7]. Blooms of *C. polykrikoides* are detrimental to the highly developed aquaculture industry in Korean coastal waters (KCW) [4,8]. The economic losses due to these dinoflagellate blooms were estimated at USD 7 million in 1993, USD 70 million in 1995, and USD 4–18.6 million per year in 2000–2003 and 2007 [4,8]. For this reason, extensive studies have been conducted to understand the precise mechanism of *C. polykrkoides* blooms [4,9,10,11,12,13,14,15,16]. However, this mechanism remains largely unknown. For example, *C. polykrikoides* blooms occurred annually from 1995 to 2007 but inexplicably decreased beginning in 2008. In 2009–2012, massive *C. polykrikoides* blooms were not observed, even though small-scale blooms were detected in some areas. Interestingly, massive blooms of *C. polykrikoides*, which covered wide areas along the south and east coasts at high densities, reoccurred in 2013. From then, these dinoflagellate blooms were continuously generated in 2014–2015 but disappeared again in 2016 [12]. Based on previous studies, physiochemical factors (e.g., temperature, salinity inorganic nutrients, etc.) can play a key role in the formation and termination of this dinoflagellate bloom in KCW [17]. However, it is unclear which factor is capable of leading to this sporadic annual pattern of the *C. polykrikoides* bloom in KCW. Thus, tracking the annual cycles of the *C. polykrikoides* bloom is thought to be an important and required long-term effort to determine the relationship between environmental factors and the bloom dynamics in KCW.

In the present study, we aimed to elucidate a potential factor that can lead to a sporadic pattern of annual blooms of *C. polykrikoides* along the Tongyeong coast, where *C. polykrikoides* blooms annually occur in southern KCW. To address this aim, we conducted a field survey along the Tongyeong coast in 2016 when this dinoflagellate bloom did not occur, unlike in previous years (2012–2015), and the environmental data in 2016 were compared with those from previous years.

## 2. Results

### 2.1. Physico-Chemical Factors along the Tongyeong Coast

**Figure 1:**
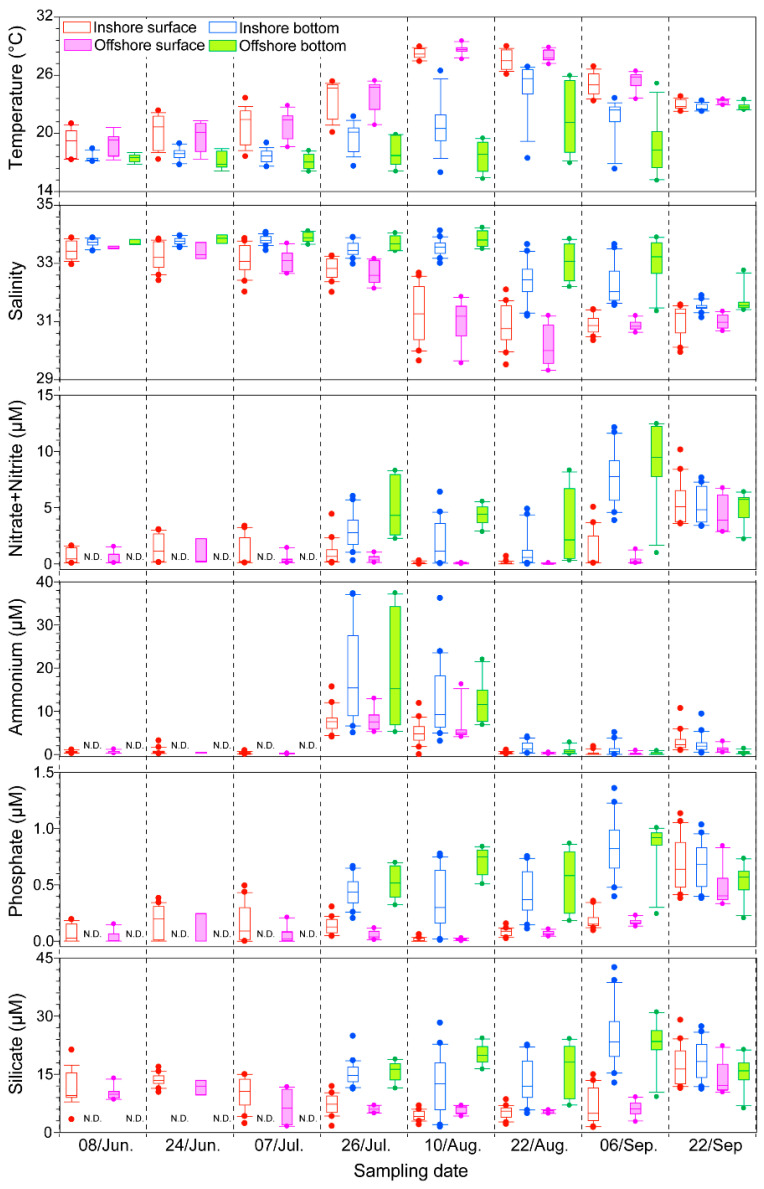
Box plots of the surface and bottom abiotic parameters in inshore and offshore waters (temperature, salinity, and nutrients). The medians are presented by solid lines, and the whiskers indicate the 10th and 90th percentiles.

The water temperature ranged from 17.3 to 29.5 °C, gradually increased from June to August, and then abruptly decreased in September (Figure 1 and Figure S1). Due to the presence of a thermocline, water stratification was observed both inshore and offshore of the Tongyeong coast during the study period, particularly in August (Figure S1). At the surface, the average offshore water temperature was generally higher than inshore, whereas the inshore water temperature was relatively higher than the temperature of the offshore waters in the bottom layer. Particularly, in August and September, the inshore and offshore water temperatures exhibited a significant difference (t-test, *p* < 0.05).

The salinity inshore (29.5 to 34.1) and offshore (29.3 to 34.2) showed similar ranges during the study period (Figure 1 and Figure S1). At the surface, the salinity slightly decreased in August and abruptly declined on the 10th of August (ca. 31.2). Then, the salinity slightly increased in September. By comparison, at the bottom, the salinity maintained higher values (above 33) until early August (10th) and then gradually dropped from the 22nd of August to September. Interestingly, unlike the usual patterns, the offshore waters had significantly lower surface salinity (t-test, *p* < 0.05) than the inshore waters from late July (26th) to August, even though there was no significant difference on the 10th of August.

The concentrations of nitrite + nitrate (0 to 2.22 μM), ammonium (0.01 to 16.27 μM), phosphate (0.01 to 1.13 μM), and silicate (1.35 to 29.0 μM) along the Tongyeong coast were investigated in this study (Figure 1 and Figure S2). During the study period, all nutrients (nitrite + nitrate, ammonium, phosphate, and silicate) showed relatively higher concentrations on the 26th of July, compared to in June and on the 7th of July. The nutrient concentrations at the bottom were generally higher than those at the surface, even though the concentrations at the bottom were not investigated from June to early July, due to the loss of the water samples. The concentrations of nitrite + nitrate, phosphate, and silicate gradually increased from the 26th of July, whereas the ammonium concentration peaked for two weeks (from 26th July to 10th August). Throughout the statistical analysis (t-test), there were no significant differences in nutrient concentrations between inshore and offshore waters.

**Figure 2:**
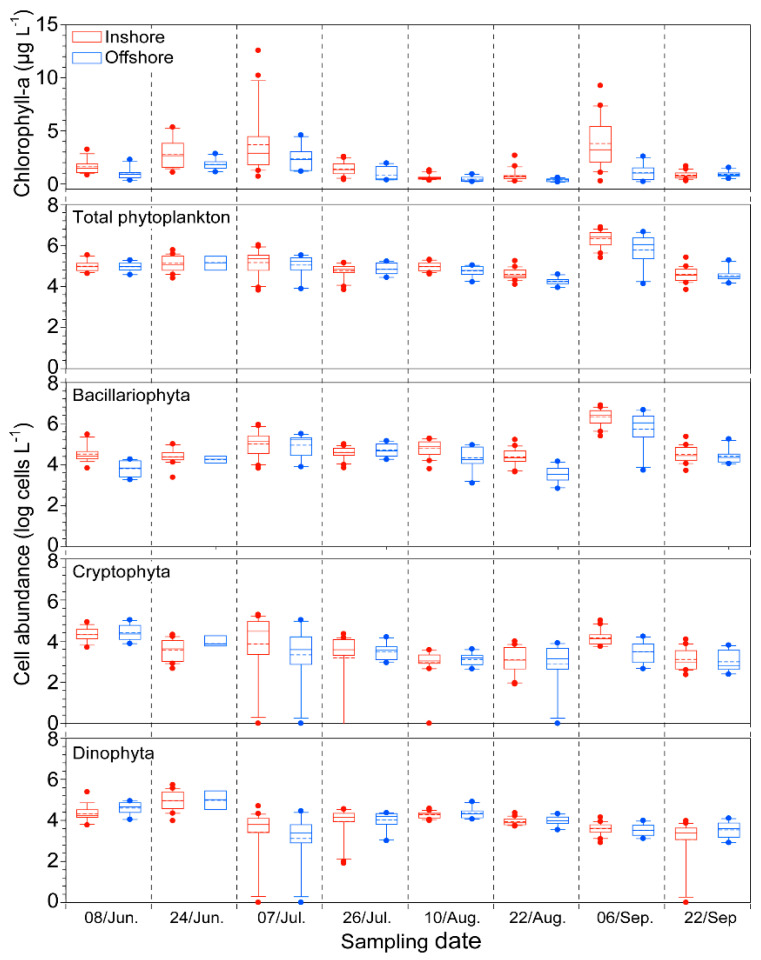
Box plots of the biotic parameters in inshore and offshore waters (Chl-*a*, total phytoplankton, bacillariophyta, cryptophyta, and dinophyta abundance). The medians and means are presented by solid lines and dotted lines, respectively, and the whiskers indicate the 10th and 90th percentiles.

During the study period, the chlorophyll *a* (Chl-*a*) concentration ranged from 0.16 to 12.6 µg L^−1^, and it peaked twice on the 7th of July and the 6th of September (Figure 2 and Figure S3). In addition, the inshore Chl-*a* concentration was significantly higher than the offshore concentration.

### 2.2. Biological Factor: Phytoplankton

**Figure 3:**
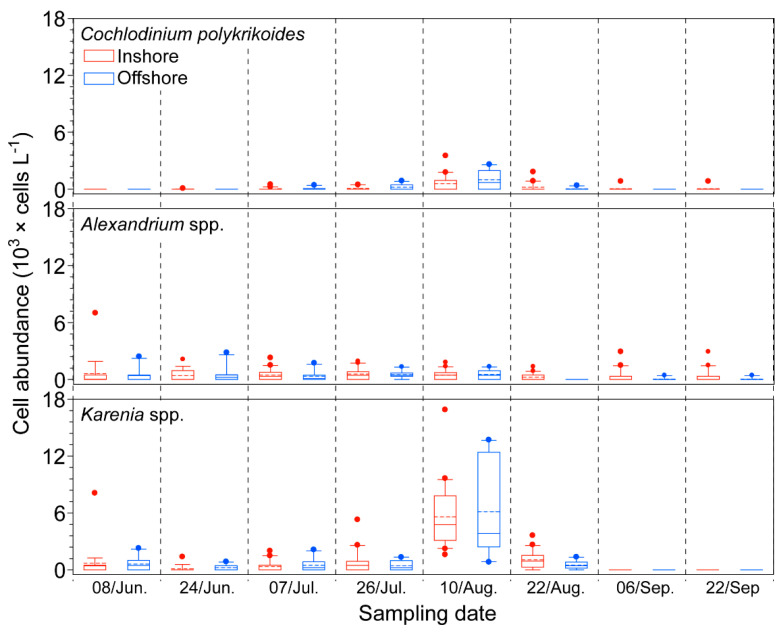
Box plots of the abundances of three dinoflagellates (top: *Cochlodinium polykrikoides*; middle: *Alexandrium* spp.; bottom: *Karenia* spp.) in inshore and offshore waters (*C. polykrikoides, Alexandrium* spp., and *Karenia* spp. abundance). The medians and means are presented by solid lines and dotted lines, respectively, and the whiskers indicate the 10th and 90th percentiles.

The abundance of phytoplankton exhibited a similar dynamic pattern as the concentration of Chl-*a*; there were two peaks of phytoplankton biomass on the 7th of July and the 6th of September, even though the first peak was not as high as the second peak (Figure 2). The inshore phytoplankton biomass (0.7 × 10^4^ to 7.8 × 10^6^ cells L^−1^) and the offshore biomass (0.8 × 10^4^ to 4.6 × 10^6^ cells L^−1^) had similar abundances, and they were composed of three dominant taxa, Bacillariophyta, Cryptophyta, and Dinophyta. Among them, Bacillariophyta was the most dominant in the inshore and offshore areas of the Tongyeong coast over the study period, and its abundance inshore was generally higher than that offshore, except on the 26th of July (Figure 2). The highest cell abundance was observed on the 6th of September in both inshore (average, 3.0 × 10^6^ cells L^−1^) and offshore (average, 1.4 × 10^6^ cells L^−1^) waters. The abundance of Cryptophyta ranged from 1.4 × 10^3^ cells L^−1^ to 4.9 × 10^4^ cells L^−1^. The Cryptophyta showed similar dynamic patterns as Bacillariophyta; the cell abundance peaked twice, on the 7th of July and the 6th of September, even though this taxon was not the most dominant taxon due to lower cell abundance than Bacillariophyta. The Dinophyta abundance ranged from 3.0 × 10^3^ cells L^−1^ to 1.7 × 10^5^ cells L^−1^. Dinophyta was the most dominant taxon on the 24th of June in both inshore and offshore waters. Interestingly, on the 22nd of August, the Dinophyta showed lower proportions than Bacillariophyta inshore, whereas it was the most dominant phytoplankton taxon offshore. A few *C. polykrikoides* cells (85 cells L^−1^) were first observed inshore on the 24th of June, but this dinoflagellate disappeared from the inshore samples that were collected on the next sampling date (7th of July) (Figure 3 and Figure S4). By comparison, in the offshore samples, a few *C. polykrikoides* cells were detected at this time, and more cells were observed further offshore (Figure S4). After the 7th of July, this dinoflagellate gradually increased, reached the highest abundance (4.2 × 10^3^ cells L^−1^) on the 10th of August, and then gradually decreased. In the Dinophyta communities, *Alexandrium* spp. and *Karenia* spp. were co-distributed when *C. polykrikoides* abundance was relatively high over the study period (from July to August) (Figure 3). The abundance of *Alexandrium* spp. ranged from 0 to 7.0 × 10^3^ cells L^−1^, and it showed two peaks in abundance on the 8th of June (average, 619 cells L^−1^) and the 10th of August (average, 534 cells L^−1^). By comparison, the abundance of *Karenia* spp. was predominantly higher than that of the other dinoflagellates in both inshore and offshore samples on the 10th of August.

### 2.3. Comparisons of Water Temperature and Salinity between the Previous Four Years and 2016

In this study, the environmental conditions along the Tongyeong coast were compared between the previous four years (2012–2015) and 2016. The ranges and averages of the water temperature and salinity in August, when *C. polykrikoides* blooms usually occurred, were investigated, and significant differences were analyzed using one-way ANOVA (Table 1). In 2016, compared to the previous years, the range of water temperature was relatively narrow (3.4 °C), and the average value was significantly distinct (one-way ANOVA; F =102; *p* < 0.001). The salinity in 2016 ranged from 29.3 to 32.7, and the lowest value (29.3) was relatively lower than that in the previous years, except in 2014. Moreover, the average salinity in 2016 was also significantly different from the salinity over the previous four years (2012–2015) (one-way ANOVA; F = 21.6; *p* < 0.001). The average value of salinity in 2014 was identical to that in 2016, but the standard deviation in 2016 was lower than that in 2014, implying that the salinity variation in 2016 was lower than that in 2014.

### 2.4. Changjiang River Discharge and Duration of Blooms

**Figure 4:**
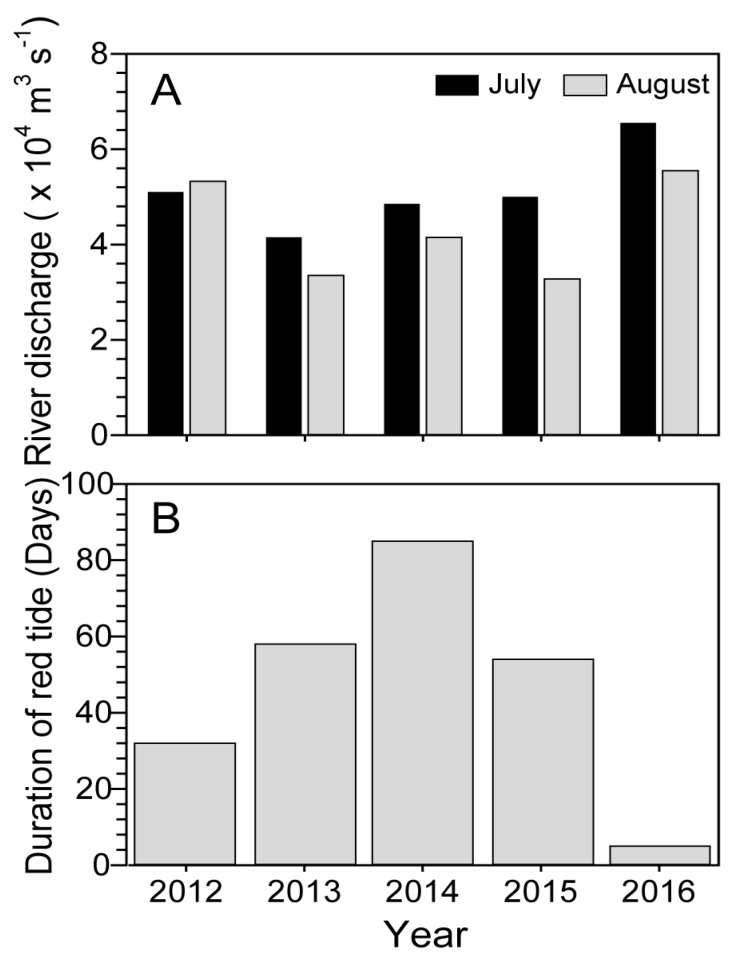
Average amount of Changjiang River discharge in July and August (**A**), and the duration of *C. polykrikoides* blooms (**B**) along the Tongyeong coast over the past five years (2012–2016). Data were obtained from the Ministry of Water Resources of the People’s Republic of China and National Institute of Fisheries Science of Korea.

Based on the data from the five years (2012–2016), the Changjiang River discharge was generally higher in July than in August (Figure 4A). The highest and second-highest amounts of discharge were observed in July of 2016 (6.6 × 10^4^ m^3^ s^−1^) and 2012 (5.1 × 10^4^ m^3^ s^−1^), respectively. In comparison, the discharge amount in 2013–2015 was relatively lower (4.2 to 5.0 × 10^4^ m^3^ s^−1^). The durations of the *C. polykrikoides* blooms along the Tongyeong coast were longer in 2013–2015, when the Changjiang River discharge was observed to be lower (Figure 4B). By comparison, the bloom duration was only four days in 2016, when the maximum amount of discharge was observed.

### 2.5. Satellite Images and Wind Direction Along the Goheung Coast

**Figure 5:**
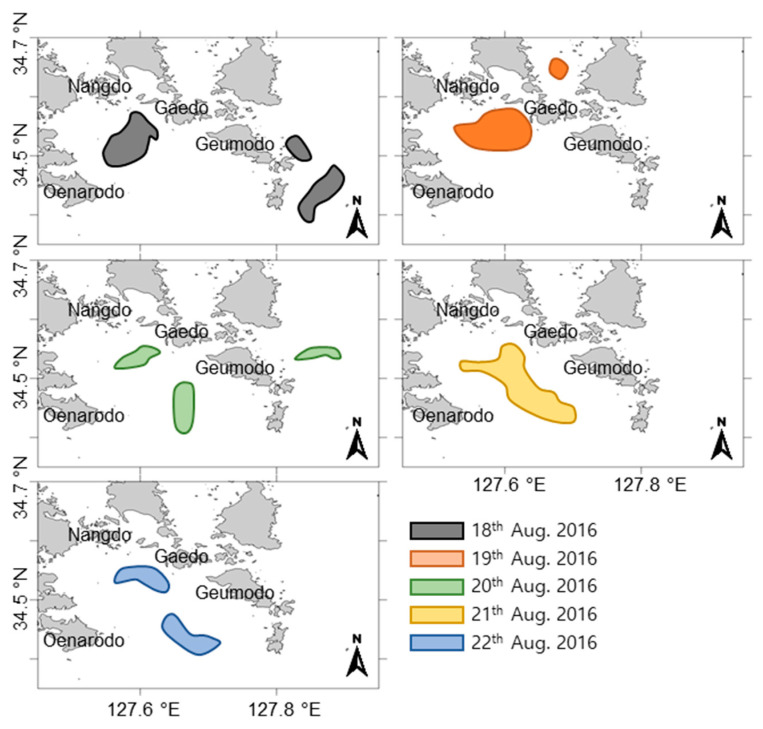
Satellite images showing the migration of *C. polykrikoides* bloom patches along the Goheung-Oenarodo coast in August 2016. Data were obtained from the Korea Institute of Ocean Science & Technology.

**Figure 6:**
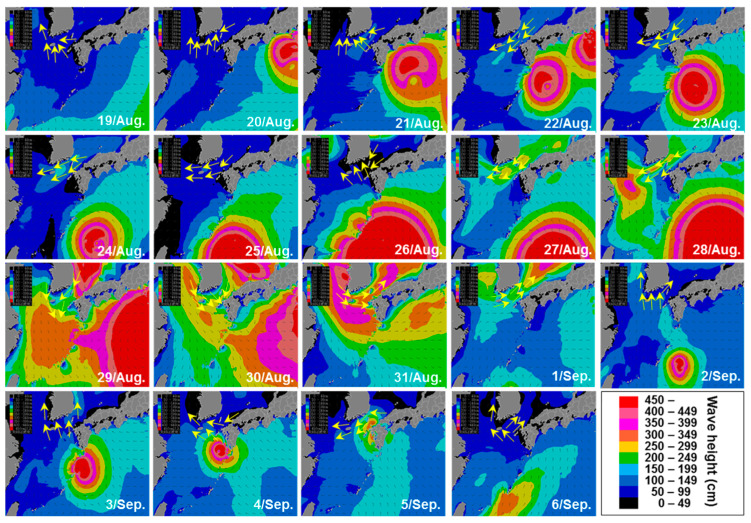
Spatial profiles of wind direction and wave height in southern Korean coast water and the East China Sea from the 16th of August to the 6th of September, 2016. Data obtained from the International Meteorological & Oceanographic Consultants Co. Ltd.

Patches of *C. polykrikoides* blooms surrounding the Goheung-Oenarodo coast were observed from satellite images in August 2016 (Figure 5). On the 18th of August, a small-scale *C. polykrikoides* patch was first observed. Then, the scales of the bloom patches gradually expanded until the 21st of August. On the 22nd of August, the *C. polykrikoides* bloom patch divided into two parts and then disappeared. In this study, the wind direction in southern KCW was investigated during the period when *C. polykrikoides* bloom patches were detected along the Goheung-Oenarodo coast. As a result, westward winds consistently occurred in southern KCW, resulting in the prohibition of eastward bloom expansion. Based on the typhoon record from the Joint Typhoon Warning Center, the typhoon Lionrock approached Kyusyu, Japan at the end of August, 2016. At that time, the wave height in the East China Sea and the western North Pacific Ocean was enhanced due to this typhoon (Figure 6). In southern KCW, the wave height was gradually increased from the 27th to 31st of August, 2016, and it ranged from 2 to 4.5 meters.

### 2.6. Statistical Analysis Using the Canonical Correspondence Analysis

**Figure 7:**
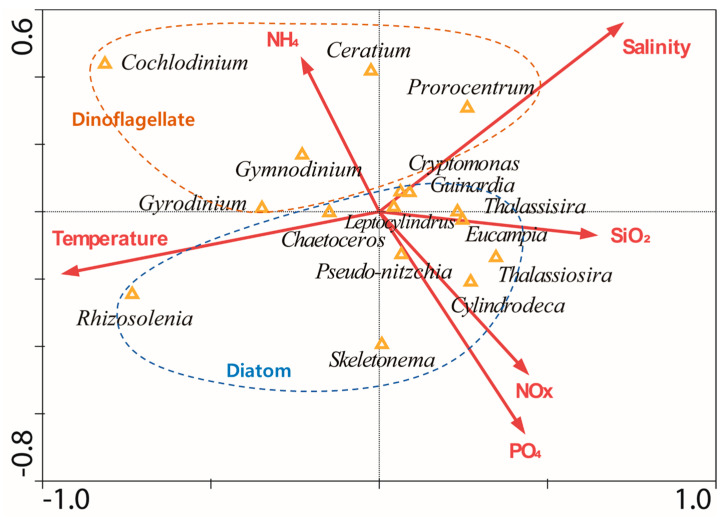
The canonical correspondence analysis (CCA) biplot of the environmental factors (arrow) and dominant phytoplankton genus (triangle).

To understand the relationships between the environmental factors and the dominant phytoplankton taxa (at the genus level), the canonical correspondence analysis (CCA) analyses were conducted using data from all surveys (Figure 7). The eigenvalues of the first and second axes were 0.072 and 0.036, respectively. In the CCA plot, dinoflagellates and diatoms had a clearly different relationship; dinoflagellates, including *C. polykrikoides*, generally had a strong positive relationship with the concentration of ammonia, whereas diatoms had a strong positive relationship with other nutrients, such as nitrite + nitrate, phosphate, and silicate.

## 3. Discussion

### 3.1. Initial Population Introduction in Southern Korean Coastal Waters

**Figure 8:**
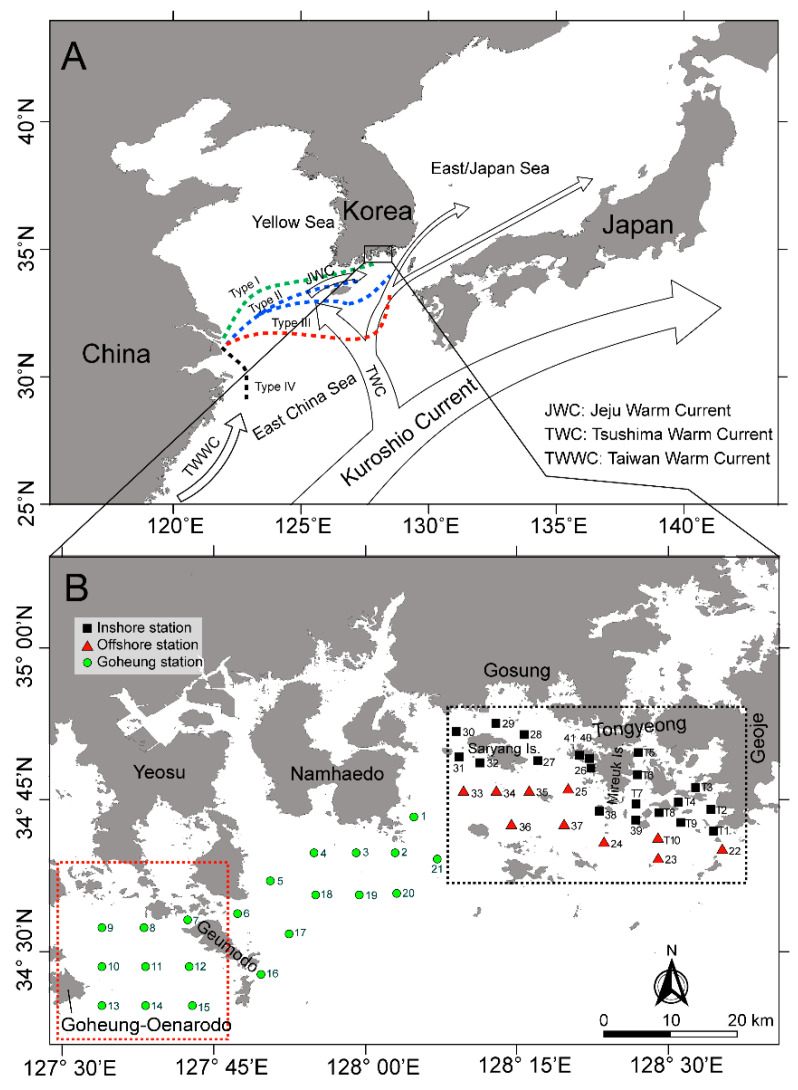
Map of the study area. (**A**) The patterns of Changjiang River discharge movement (each dashed line shows four different pathways of the Changjiang River discharge); (**B**) sampling sites. The stations in the dashed black box are the biweekly survey stations located inshore (black square) and offshore (red triangle), whereas the stations represented by green circles were collected once after the termination of the *C. polykrikoides* blooms in the Goheung-Namhaedo area. The area in the red dashed box is the Goheung-Oenarodo coast.

The initial introduction of viable vegetative *C. polykrikoides* cells in the water column can play a crucial role in the development of the bloom, and understanding the introduction mechanisms is highly necessary to clarify the bloom mechanisms. In KCW, *C. polykrikoides* blooms are generally initiated near the Goheung-Oenarodo area. Based on previous findings [4,11,12,18], this accumulation may be affected by a combination of multiple factors as follows: (i) geographical features with semi-closed bays between Narodo and Guemodo Island (Figure 8B) and an inflow of the initial population from offshore waters into the Narodo-Goheung area via water currents, (ii) thermohaline fronts formed between southern KCW and the Tsushima Warm Current (TWC), (iii) a salinity front formed between inshore and offshore waters caused by diluted water from the Changjiang River in China, and (iv) dominant seasonal south winds toward the Korean peninsula in the summer. In addition, the germination of the seed population (e.g., resting cysts) can also contribute to the introduction of *C. polykrikoides* cells into the water column. In recent studies, this dinoflagellate was found to produce resting cysts and was distributed throughout southern KCW [19,20]. However, there is a little doubt that the germination of this seed population can initiate a bloom in the KCW. For example, resting cysts of *C. polykrikoides* were only observed in one region (Tongyeong coast), despite the occurrence of this dinoflagellate bloom throughout southern KCW. Moreover, as measured by the quantification analyses via both microscopic and molecular assays, the abundance of resting cysts in the sediments was extremely low [20,21]. In this study, a few *C. polykrikoides* cells were first observed inshore along the Tongyeong coast on the 7th of July, but it was unlikely that this population contributed to the initiation of the blooms (Figure 3 and Figure S4). By contrast, several *C. polykrikoides* cells were detected offshore during this time. More cells were observed further offshore, implying that this dinoflagellate is likely introduced from the outer region. Moreover, in the Goheung-Oenarodo area, the *C. polykrikoides* population was observed to be 300~5000 cells L^−1^ on the 30th of July, 2016 [22], which was associated with the continuous accumulation of cells combined with the input of *C. polykrikoides* cells via the inshore and alongshore mixing of waters containing high densities of *C. polykrikoides* cells. Following the month of the initial *C. polykrikoides* cell observations, the first *C. polykrikoides* bloom patches were detected on the 20th of August by satellite ocean color observations (Figure 5). At that time, the density of *C. polykrikoides* ranged from 3 × 10^5^ to 5 × 10^6^ cells L^−1^ near the Goheung-Oenarodo area. Based on previous findings, during the summer (July–August), the large-scale transport of algal blooms containing *C. polykrikoides* species by the TWC was confirmed by satellite Chl-*a* images [23,24], indicating that *C. polykrikoides* can be transported long distances by the warm current from the East China Sea (ECS) [12,25,26,27]. Moreover, recent studies [13,15,28] provide genetic evidence that is suggestive of this long-distance transfer using microsatellite markers and ribotype-specific real-time PCR. Therefore, these findings suggest that the cell accumulation by hydrographically south-to-north flow from the ECS linked with the TWC can play an important role in bloom initiation in southern KCW. Generally, this small patch of bloom used to expand to eastern areas (especially along the Tongyeong coast), resulting in a massive *C. polykrikoides* bloom throughout the entire southern KCW. However, in 2016, a massive bloom did not occur due to the dominating east-to-west flow of the surface currents and the combination of unfavorable conditions (e.g., high water temperature, low salinity, and typhoon events), as described below.

### 3.2. Effect of Environmental Factors on the Inhibition of Cochlodinium Bloom Formation

Water temperature and salinity are widely recognized as major factors that directly affect the dynamics of phytoplankton. It is widely known that *C. polykrikoides* has a relatively wider tolerance for physico-chemical factors, such as water temperature and salinity, than other phytoplankton species [29]. However, both laboratory and field observations [26,30,31,32] have shown that the optimum temperature for *C. polykrikoides* growth ranged from 23 to 27 °C, indicating that those temperature ranges are suitable for *C. polykrikoides* blooms, even though some blooms have occasionally been generated under lower water temperatures (14–16 °C). In this study, the relatively higher abundance of *C. polykrikoides* cells was observed on the 10th of August, but the abundance of this dinoflagellate abruptly decreased on the 22nd of August along the Tongyeong coasts, including the inshore and offshore waters (Figure 3 and Figure S4). At that time in 2016, strong water stratification was generated, and the surface water temperature was abnormally high (c.a., 30 °C). Interestingly, the water temperature in August 2016 was significantly higher than in previous years (*p* < 0.001) (Table 1). Given these findings, the growth of the *C. polykrikoides* population, which has a relatively narrow optimum temperature range (c.a. 23–27 °C), may be inhibited by abnormally higher water temperatures (30 °C), thereby reducing the chance to generate this dinoflagellate bloom in 2016.

Based on laboratory observations, the optimum salinity for *C. polykrikoides*, which belongs to the East Asian ribotype, ranged from 32 to 34.4, even though the species has been found to have wide tolerances for salinity (25 to 40) and water temperature [30,31]. Moreover, in field observations, this dinoflagellate generated blooms under relatively high salinities (32–34.5) that were almost identical to the optimum salinity range determined by laboratory observations [12,26,31]. These findings indicate that the optimum salinity can be a crucial factor for the initiation and maintenance of the *C. polykrikoides* bloom in the field. During the study period, a higher salinity (≥32) was consistently observed in both inshore and offshore waters of the Tongyeong coast until July (Figure 1). However, it was not only decreased on the 10th of August, abruptly, but also maintained a lower value in August (30.9 ± 0.86) (Figure 1). In comparison with the results for the previous five years (Table 1), the average salinity in 2016 (August) was significantly distinct from that in the previous four years (2012–2015). Although the average of salinity in 2016 was identical to that in 2014, the salinity variation in 2016 (0.86) was two-times lower than that in 2014 (1.97). This indicates that the salinity in August, 2016 was consistently lower than the optimum salinity range (32–34.5) for *C. polykrikoides* growth, unlike that in 2014. Interestingly, the salinities of the inshore and offshore waters simultaneously decreased, and the offshore surface salinity was significantly lower than the inshore salinity on the 22nd of August (t-test; t = 2.25, *p* < 0.05). A large volume of runoff from Changjiang River discharge at Datong (China) in the summer strongly influenced the Chinese coastal water and was especially prevalent during the summer because of extensive precipitation runoff from China [33,34]. According to Bai et al. (2014) [34], there were three types of plume shapes as follows (dashed lines in Figure 8A): Type I: most of the plume water crossed the Jeju Strait into the Korea-Tsushima Straits with only a small fraction remaining on the shelf of the ECS, Type II: the plume water expanded northward along the China coast and then turned eastward through the Jeju Strait, and Type III: the plume front moved southeastward, which is a rare case in the summer. It has been suggested that the low-salinity plume patches such as Types I and II have significantly influenced southern KCW and the Kita-Kyusyu coast in Japan [34,35]. Recently, Wei et al. (2015) [36] demonstrated that Changjiang diluted water greatly influences southern KCW via the advancement and recession of the Taiwan Warm Current, resulting in a reduction in salinity level in southern KCW. The diluted water especially influences the inner branch and the Kuroshio intrusion of the TWC, and this diluted water approaches southern KCW with a time lag of 1 to 2 months [24,34]. In July 2016, a large amount of discharge (>6.5 × 10^4^ m^3^ s^−1^ in July) from the Changjiang River was observed, and this discharge continuously occurred in August even though the amount of discharge slightly decreased (Figure 4A). To better understand the effect of the lowered salinity level due to Changjiang diluted water on bloom termination, statistical correlation analysis was conducted using the data for salinity and *C. polykrikoides* abundance in August when salinity in southern KCW was decreased due to the introduction of Changjiang diluted water. As a result, *C. polykrikoides* abundance was significantly related to the salinity level (Pearson correlation, r = 0.989; *p* < 0.05). Given these findings, a large amount of discharge from the Changjiang River in July may have reduced the salinity along the Tongyeong coast in August with a time lag of one month, and this lower salinity level may have negatively affected the bloom formation of *C. polykrikoides* in 2016. Interestingly, it is still controversial whether or not Changjiang River discharge is capable of mitigating *C. polykrikoides* blooms in KCW. Based on a previous study [12], the Changjiang River discharge can induce an increase in nutrient concentrations in southern KCW, resulting in a positive effect on the formation of *C. polykrikoides* blooms. Therefore, further attention should be paid to the actual effect of Changjiang River discharge on the formation of *C. polykrikoides* blooms in southern KCW.

As competitors for nutrients, central diatoms theoretically have an advantage over dinoflagellates. The growth rates of diatoms are generally higher than those of dinoflagellates under the same environmental conditions, as previously demonstrated by Eppley (1969) [26], Elbrächter (1977) [37], and Smayda (1997) [3]. Indeed, elevated nutrients and lower salinity from freshwater runoff after rainfall are likely to favor diatoms over the dinoflagellates and rapidophyta [17,38,39,40]. In the present study, under the continuously low nutrients in the euphotic surface layer, the dinoflagellates and crytophyta species were relatively dominant on the 8th and 24th of June and were composed of *Prorocentrum* and *Ceratium* species. By comparison, after rainfall (on the 7th of July), the relatively high Chl-*a* concentrations are considered to be attributed to the diatoms that appear under the high nutrient levels and slightly lower salinities inshore, compared to in offshore waters (Figure 2, Figures S3 and S5). At that time, the dominant diatoms were *Chaetoceros* spp., *Psudo-nitzschia* spp., and *Hemiaulus* spp., which composed more than ca. 70% of the community at most stations. On the 26th of July, *Chaetoceros decipiens* and *C. affinis* were present in the inshore area, and *Chaetoceros compressus* was relatively dominant in the offshore area (data not shown). In August, not only did the abundances of the *Alexandrium* spp., *Cochlodinium* spp., and *Karenia* spp. dinoflagellates peak but they were also dominant in the offshore area, implying that these dinoflagellates may have competitive advantages. These species may adapt to lower nutrient conditions through their nutrient uptake physiological characteristics and may have an ecological advantage due to their diel vertical migration activities (Figure 1, Figure 2, Figure 3 and Figure S2). During this time, *Karenia* spp. were predominantly more abundant compared to *Alexandrium* spp. and *Cochlodinium* spp. Although there is no direct evidence for an ecological relationship among these dinoflagellates, they are likely to have similar life forms (Type IV, frontal zone taxa described in Smayda (2002) [41]) and habitat preferences, suggesting that they may have a competitive relationship. Therefore, the higher abundance of *Karenia* spp. may have contributed to the inhibition of *C. polykrikoides* bloom formation. However, to better understand the ecological relationship among these dinoflagellates, further studies, such as physiological experiments, are necessary.

Indeed, when co-occurring diatoms such as *Pseudo-nitzschia* spp. and *Chaetoceros* spp. were abundant, the dinoflagellate *C. polykrikoides* was constantly kept at a low density, implying that the *C. polykrikoides* population was not well adapted to the diatom-dominated ecosystem (Figure 3 and Figure S4). Lim et al. (2014) [7] demonstrated that the significant development of diatoms could inhibit the growth rate and swimming speed of *C. polykrikoides*, which adversely influences and reduces the depths reached by *C. polykrikoides* through diel vertical migration. As noted by Smayda (1997) [3] and Anderson et al. (2005) [42], chain-forming dinoflagellates such as *Cochlodinium* and *Alexandrium* have adaptive advantages in terms of swimming speed and their ability to withstand vertical velocities. Improved swimming abilities may provide some photosynthetic advantages [9] and allow access to the deep nutricline or sediments at night [3,43,44,45]. Furthermore, during the study period, the water was consistently stratified along the Tongyeong coast, which resulted in a negative effect on the vertical migration of *C. polykrikoides* under the diatom-dominated conditions of the stratified upper layer. These findings indicate that the reduction in the swimming speed of *C. polykrikoides* due to dense diatom populations and stratification prevented the migration of *C. polykrikoides* to the bottom where nutrient concentrations are high. In addition, the presence of dense diatoms in July and early August has a negative effect on the growth of *C. polykrikoides*. Therefore, these conditions are likely to delay or prevent the bloom formation of *C. polykrikoides* in southern KCW, particularly along the Tongyeong coasts.

### 3.3. Extinction of the C. polykrikoides Bloom by a Typhoon Carrying Strong Winds and Heavy Rainfall

In temperate coastal regions, the passage of typhoons causes physical disturbances including vertical mixing, terrestrial runoff, and sediment resuspension [46,47]. Typhoons have significantly altered aquatic ecosystems with their powerful winds and rainfall [48,49]. In the southern ECS and KCW, these typhoons not only induce changes in the dominant phytoplankton group from flagellates to diatoms [50,51] but also contribute to the termination of *C. polykrikoides* blooms [52,53]. Lim et al. (2015) [45] demonstrated that typhoons affected the dynamics of *Cochlodinium* blooms differently, and the effect depended on the daily maximum wind speed generated by the typhoon as follows: (i) when the daily maximum wind speeds were >14 m s^−1^, *C. polykrikoides* blooms could be eliminated; (ii) if the daily maximum wind speeds remained between 5–14 m s^−1^, a significantly lower abundance of *C. polykrikoides* could be maintained. However, when the daily maximum wind speeds were <5 m s^−1^, damage to the *C. polykrikoides* cells did not occur. Between August 29 and 30 in 2016, the typhoon Lionrock approached Kyusyu, Japan. The typhoon generated strong winds with maximum speeds of 17.8 m s^−1^ along the Goheung and Tongyeong coasts (Table S1). The cores of the typhoons were positioned within 300 km of the coast, which directly affected the KCW, even after the typhoon passed Japan. Based on the satellite image data, patches of *C. polykrikoides* blooms were clearly observed in southern KCW until 22nd of August (Figure S4), and this dinoflagellate bloom consistently maintained right before the typhoon passed through these areas [22]. To examine the effects of the typhoon on the growth of *C. polykrikoides*, the cell density of this dinoflagellate was investigated via light microscopy at 22 stations along the Goheung coast after the typhoon passed (7th of September). As a result, cells of *C. polykrikoides* were not detected (data not shown). In addition, the wave heights were elevated in southern KCW due to the typhoon event (Figure 6). Consequently, there were no significant differences in the water temperature or salinity between the surface and bottom layers, indicating that a well-mixed water column occurred over a 15 m depth in the Goheung-Oenarodo area (Figure S6). Given these findings, the typhoon may play an important role in the termination of *C. polykrikoides* blooms in southern KCW.

## 4. Conclusions

This study was conducted to characterize which factor(s) could cause the disappearance of the *C. polykrikoides* bloom in southern KCW in 2016. To summarize, (i) a larger than average amount of Changjiang River discharge was observed in July, 2016, and the Changjiang diluted water approached the Tongyeong coasts 1 month after the discharge, which resulted in abnormally low salinity and higher water temperatures in the offshore waters in August; (ii) the surface water currents driven by the westward wind prohibited the eastward expansion of the *C. polykrikoides* blooms along the Goheung-Oenarodo coast; (iii) a typhoon that resulted in strong winds and heavy rainfall may have induced the decline of this dinoflagellate bloom in late August; and (iv) the higher abundance of competitive phytoplankton species may have negatively affected *C. polykrikoides* growth. Given these findings, this study provides a deeper understanding of the dynamics of the *C. polykrikoides* bloom surrounding the Tongyeong coast in 2016.

## 5. Materials and Methods

### 5.1. Study Area

The Tongyeong and Goheung-Oenarodo coasts are centrally located in southern KCW. Based on data over the past 20 years, *C. polykrikoides* blooms are generally initiated along the Goheung-Oenarodo coast. Due to the accumulation of cells by the currents, these dinoflagellate blooms may spread into other coastal areas, such as the eastern part of southern KCW, the Yellow Sea, and the East Sea (Sea of Japan) [4]. The southern Korean coastal area is geographically affected by ocean currents including the Jeju Warm Current (JWC) and the Tsushima Warm Current (TWC), which originate from the outer regions, particularly in the summer [35,54]. Thus, the physico-chemical conditions between the KCW and the outer regions are closely linked. For example, the Changjiang River in China occasionally releases a large amount of discharge, resulting in the reduction of surface salinity in the East China Sea (ECS) [24,34,55], and this environmental variation can influence the KCW via the combination of the JWC, the TWC, and the Taiwan Warm water current (TWWC) (Figure 8A).

### 5.2. Field Survey

The field sampling in the Tongyeong area was carried out biweekly from June to September 2016 (Figure 8B and Table S2). To investigate the association between the termination of *C. polykrikoides* blooms and water mixing by strong winds after a typhoon, we conducted a single sampling in the coastal waters of the Goheung-Namhaedo area on the 7th of September. The sampling included water from the Goheung-Oenarodo coast, where a small-scale *C. polykrikoides* bloom was generated in August 2016. At all stations during the field sampling, the vertical profiles of the biophysical parameters of the seawater—such as the temperature, salinity, and fluorescence—were determined using an in situ conductivity-temperature-depth sensor (CTD; Ocean Seven 319; Idronaut Co., Brugherio, Italy). The water samples were collected at the surface with a bucket and from the bottom (1 m above the bottom) layers using a 5-L PVC Niskin sampler (General Oceanics, Miami, FL, USA). To analyze the concentration of inorganic nutrients and Chl-*a*, 0.5 L of the water samples were immediately filtered (GF/F; 47 mm; Whatman, Middlesex, U.K.) onboard the research vessel, placed in acid-cleaned polyethylene bottles, and poisoned with HgCl_2_. Then, the filters and filtrates were stored at −20 °C in dark conditions until further laboratory analysis. The concentrations of inorganic nutrients (ammonia, nitrate, nitrite, phosphate, and silicate) were measured using a flow injection autoanalyzer (QuikChem 8000; Lachat Instruments, Loveland, CO, USA) and calibrated using standard brine solutions (RMNS, KANSO Technos Co., Ltd., Japan). The Chl-*a* concentrations were measured using a Turner-designed fluorometer (Turner BioSystems, Sunnyvale, CA, USA) following the extraction of the filtered material with 90% acetone for 24 h in the dark. To identify and enumerate the phytoplankton population, the water samples (0.5 L) were fixed with 0.5% Lugol’s solution and stored at 4 °C. These preserved samples were transferred into Sedgewick Rafter chambers after cell concentration via decanting the supernatant as described by Sournia (1978) [56]. The phytoplankton cells were counted and identified using light microscopy at 200x magnification. After counting the cells, all data were converted to obtain the phytoplankton density by considering the measured volume and concentration factors.

### 5.3. In Situ Data (Meteorological Data and River Discharge Data)

To investigate the effects of environment factors on the dynamics of *C. polykrikoides*, meteorological data from southern KCW were collected; (i) satellite images were downloaded from the Korea Ocean Satellite Center of Korea Institute of Ocean Science & Technology (KOSC; http://kosc.kiost.ac), (ii) rainfall data were obtained from the Korea Meteorological Administration (KMA; http://web.kma.go.kr), (iii) the wind direction and wave height from the Korean peninsula to southern Japan were provided by the International Meteorological & Oceanographic Consultants Co. Ltd. (http://www.imocwx.com), and (iv) typhoon records were downloaded from the Joint Typhoon Warning Center (http://weather.unisys.com/hurricane/w_pacific). In addition, previous data on the *C. polykrikoides* bloom were obtained from the National Institute of Fisheries Science of the Ministry of Oceans and Fisheries, Korea (http://www.nifs.go.kr). The Changjiang River discharge data were obtained from the Ministry of Water Resources of the People’s Republic of China website (http://www.hydroinfo.gov.cn), and these data were measured at the Datong hydrological gauge station without tidal influence, which is located 624 km upstream from the river mouth.

### 5.4. Statistical Analysis

The field data for the inshore and offshore stations were analyzed using a t-test to compare the biotic and abiotic factors between the inshore and offshore stations. Differences were considered significant at the *p* < 0.05 level. One-way ANOVA followed by Tukey’s test was used to examine the significant differences in water temperature and salinity among the previous five years (2012–2016) along the Tongyeong coast. All statistical analyses were performed using SPSS version 17.0 (SPSS, Inc., Chicago, IL, USA). The relationship of the dominant phytoplankton species with the physical variables was analyzed using canonical correspondence analysis (CCA), using the Canoco software for Windows 4.5.

## Figures and Tables

**Table 1:** The ranges and averages of the water temperature and salinity observed along the Tongyeong coast over the past five years (every August, 2012–2016). One-way ANOVA was conducted to determine statistically significant differences among the five years. Data were provided by National Institute of Fisheries Science of Korea.

		Year					*F*
		2012	2013	2014	2015	2016	
Temperature (°C)	Range Mean ± SD	24.5–27.1 (25.9 ± 0.81) ^b^	17.7–27.4 (21.8 ± 2.56) ^d^	22.8–25.1 (23.9 ± 0.69) ^c^	21.5–27.7 (24.5 ± 1.58) ^c^	26.1–29.5 (28.0 ± 0.81) ^a^	102 ***
Salinity	Range Mean ± SD	31.8–32.9 (32.4 ± 0.31) ^a^	32.0–33.8 (33.1 ± 0.47) ^a^	24.1–32.7 (30.1 ± 1.97) ^c^	31.9–33.0 (32.5 ± 0.38) ^a^	29.3–32.7 (30.9 ± 0.86) ^b^	21.6 ***

Data show the mean ± SD. Letters (a, b, c, and d) represent significant differences. ***: *p* < 0.001, SD indicates standard deviation (*n* = 18).

## Supplementary Materials

The following are available online at https://www.mdpi.com/2072-6651/12/6/390/s1, Table S1: The direction and maximum speed of wind in Goheung area from the 18th of August to the 6th of September, 2016, Table S2: List of stations used in the study, with latitude, longitude and section information, Figure S1: The spatial profiles of water temperature (A) and salinity (B) in inshore and offshore waters of the Tongyeong coast, Figure S2: Spatial profiles (at the surface) of inorganic nutrient concentrations in the inshore and offshore waters of the Tongyeong coast. A: nitrite+nitrate, B: ammonium, C: phosphate, and D: silicate, Figure S3: The spatial profiles of chlorophyll-a concentrations in the inshore and offshore waters of the Tongyeong coast. A: the surface layer and B: the bottom layer. Samples from the bottom layer were not collected from the 8th of June to the 7th of July, Figure S4: Spatial profiles (at the surface) of Cochlodinium polykrikoides abundance in the inshore and offshore waters of the Tongyeong coast from the 8th of June to the 22nd of September, 2016, Figure S5: The 10-day accumulated precipitation before each field survey (A: Tongyeong, B: Namhae, and C: Goheung coasts). These data were obtained from the Korea Meteorological Administration. Light gray background indicates the time, when C. polykrikoides blooms were terminated in southern Korean coastal waters (KCW) due to typhoon events, Figure S6: Spatial profiles (surface and bottom layers) of the environmental factors in the Goheung-Namhaedo area on the 7th of September. A: water temperature, B: salinity, C: chlorophyll-a, D: nitrite+nitrate; E: ammonium, F: phosphate, and G: silicate.
