# Supplementary material for: Potential Cause of Decrease in Bloom Events of the Harmful Dinoflagellate Cochlodinium polykrikoides in Southern Korean Coastal Waters in 2016
Source: Toxins (Basel). 2020 Jun 12;12(6):390. doi: 10.3390/toxins12060390 (PMC7374724; doi:10.3390/toxins12060390)

Supplementary Material: Potential Cause of Decrease in Bloom Events of the Harmful Dinoflagellate Cochlodinium polykrikoides in Southern Korean Coastal Waters in 2016

Seung Ho Baek, Yunji Kim, Minji Lee, Chi-Yong Ahn, Kyung Hwa Cho and Bum Soo Park

Table S1. The direction and maximum speed of wind in Goheung area from the 18th of August to the 6th of September, 2016.

Date	Wind direction	Maximum wind speed (m s^{-1})
August 18	SSE	6.3
August 19	S	4.9
August 20	S	5.3
August 21	NE	6.7
August 22	SSW	5.6
August 23	SSE	6.0
August 24	SSE	6.8
August 25	SSW	5.7
August 26	SE	10.0
August 27	E	8.5
August 28	NW	6.2
August 29	NNW	9.2
August 30	NW	17.8
August 31	W	15.5
September 1	S	8.1
September 2	SSE	6.1
September 3	NW	6.0
September 4	NE	5.6
September 5	W	5.1
September 6	NW	3.5

Table. S2. List of stations used in the study, with latitude, longitude and section information.

Station	Longitude (°N)	Latitude (°E)	Section	Station	Longitude (°N)	Latitude (°E)	Section
1	128.079	34.722	Goheung	23	128.481	34.652	Offshore
2	128.048	34.663	Goheung	24	128.392	34.679	Offshore
3	127.984	34.663	Goheung	25	128.333	34.767	Offshore
4	127.915	34.663	Goheung	26	128.368	34.805	Inshore
5	127.843	34.617	Goheung	27	128.283	34.815	Inshore
6	127.789	34.563	Goheung	28	128.260	34.858	Inshore
7	127.707	34.553	Goheung	29	128.214	34.877	Inshore
8	127.635	34.540	Goheung	30	128.149	34.862	Inshore
9	127.566	34.540	Goheung	31	128.154	34.820	Inshore
10	127.566	34.476	Goheung	32	128.187	34.812	Inshore
11	127.638	34.476	Goheung	33	128.161	34.763	Offshore
12	127.710	34.476	Goheung	34	128.215	34.763	Offshore
13	127.566	34.411	Goheung	35	128.269	34.763	Offshore
14	127.638	34.411	Goheung	36	128.239	34.708	Offshore
15	127.715	34.411	Goheung	37	128.326	34.708	Offshore
16	127.828	34.463	Goheung	38	128.384	34.731	Inshore
17	127.874	34.529	Goheung	39	128.444	34.716	Inshore
18	127.918	34.594	Goheung	40	128.365	34.817	Inshore
19	127.989	34.594	Goheung	41	128.352	34.822	Inshore
20	128.051	34.596	Goheung	T-1	128.572	34.699	Inshore
21	128.118	34.653	Goheung	T-2	128.568	34.734	Inshore
22	128.586	34.667	Goheung	T-3	128.543	34.771	Inshore
				T-4	128.514	34.747	Inshore
				T-5	128.449	34.828	Inshore
				T-6	128.447	34.791	Inshore
				T-7	128.445	34.743	Inshore
				T-8	128.482	34.729	Inshore
				T-9	128.518	34.712	Inshore
				T-10	128.481	34.686	Offshore

Figure S1. The spatial profiles of water temperature (A) and salinity (B) in inshore and offshore waters of the Tongyeong coast.

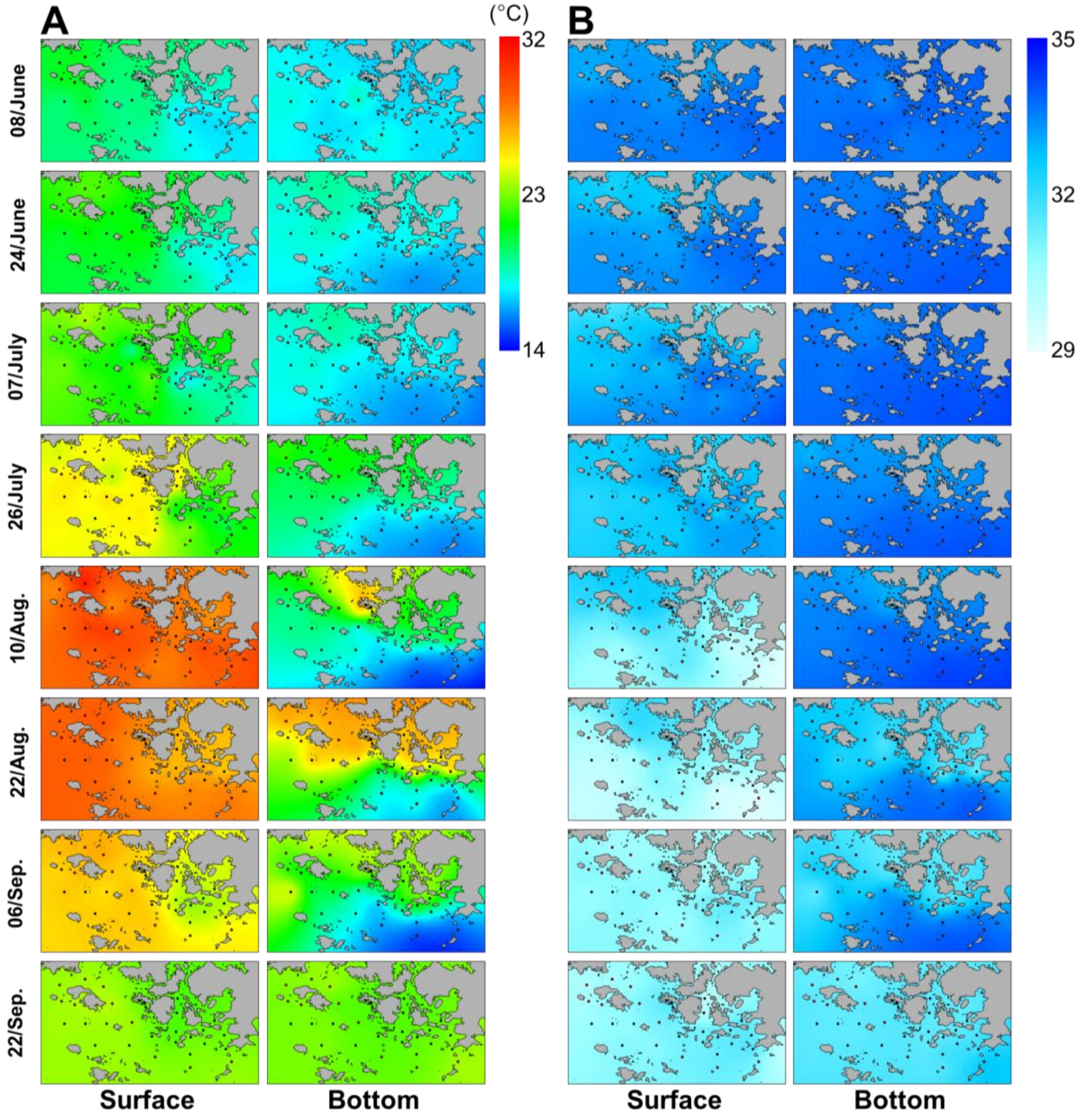

Figure S2. Spatial profiles (at the surface) of inorganic nutrient concentrations in the inshore and offshore waters of the Tongyeong coast. A: nitrite+nitrate, B: ammonium, C: phosphate, and D: silicate.

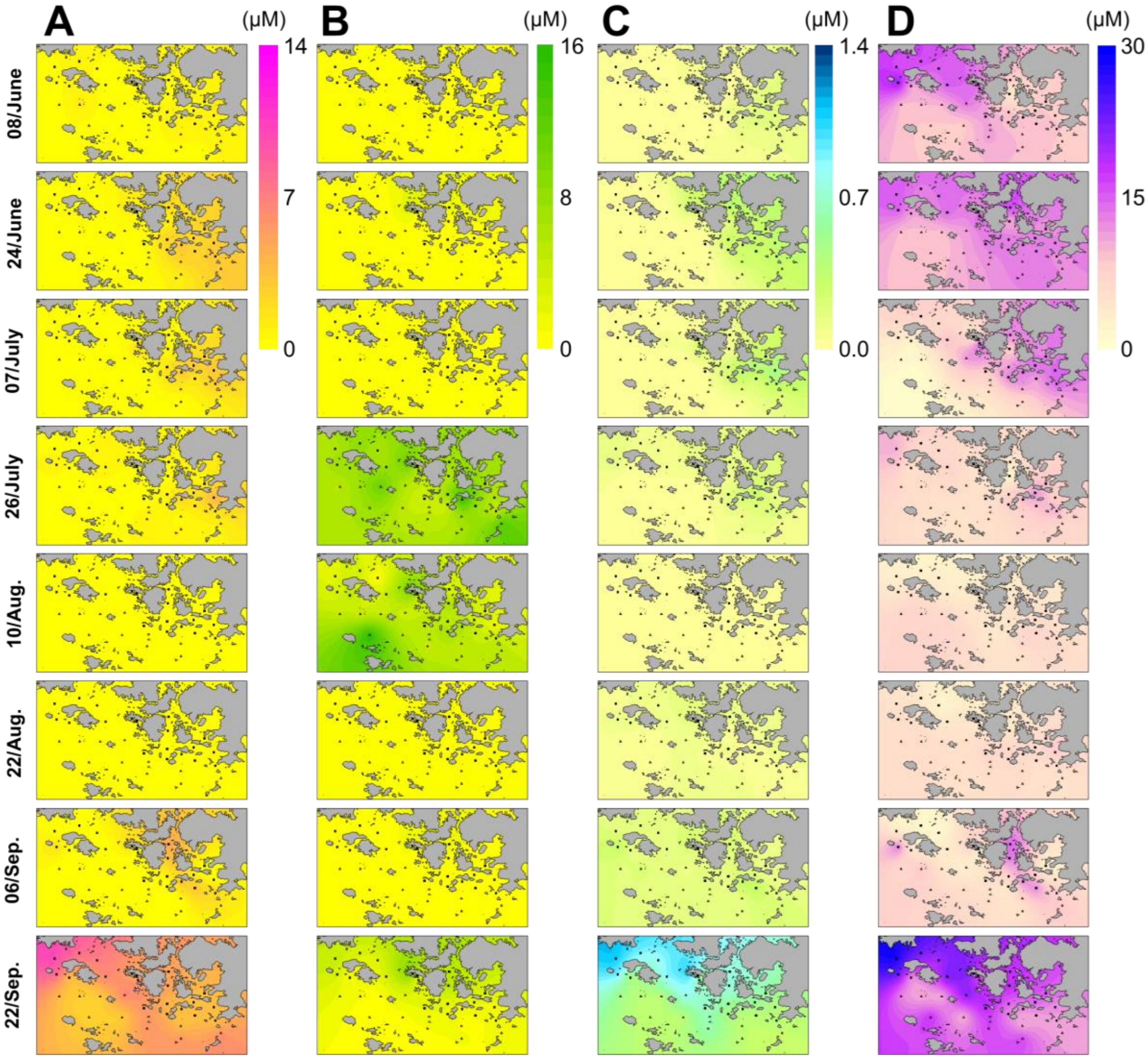

Figure S3. The spatial profiles of chlorophyll-*a* concentrations in the inshore and offshore waters of the Tongyeong coast. A: the surface layer and B: the bottom layer. Samples from the bottom layer were not collected from the 8th of June to the 7th of July.

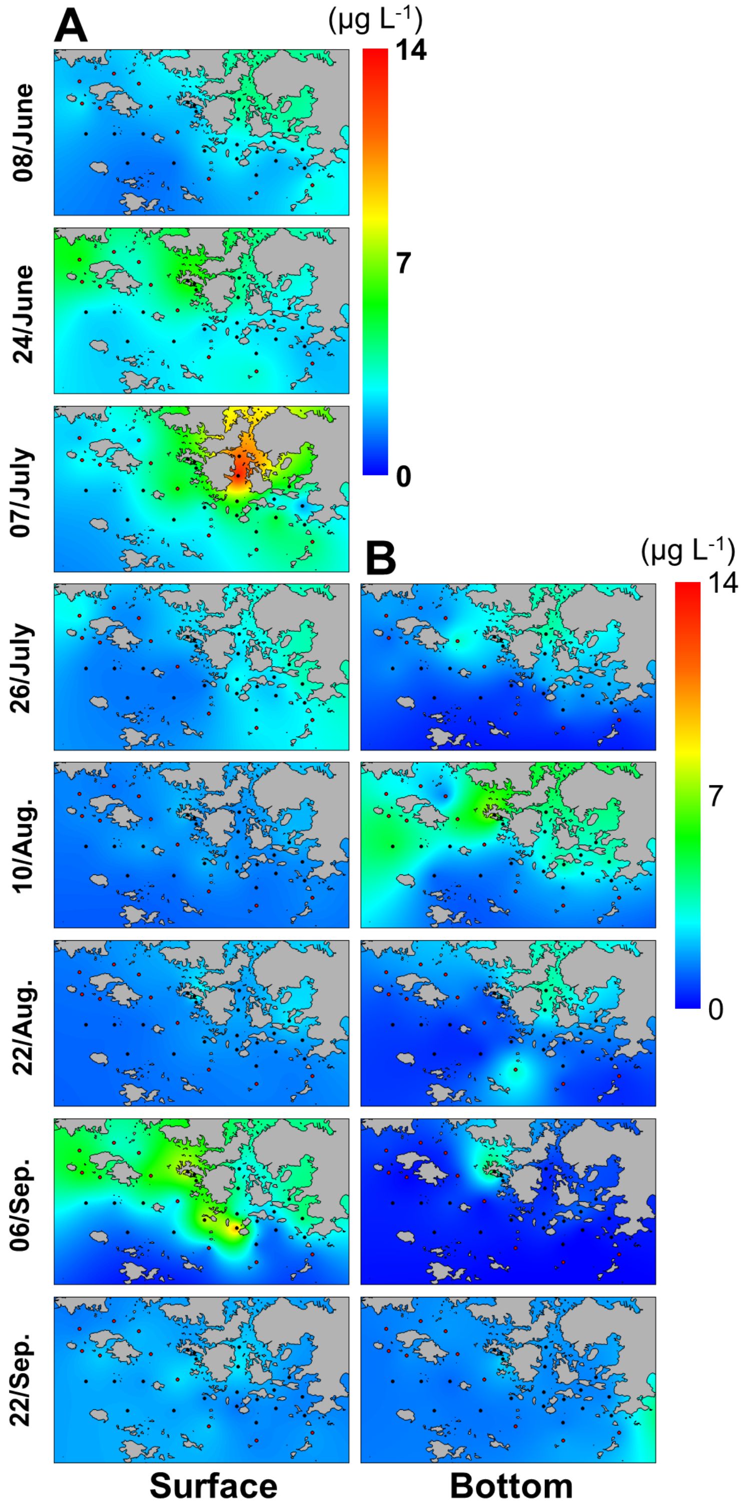

Figure S4. Spatial profiles (at the surface) of *Cochlodinium polykrikoides* abundance in the inshore and offshore waters of the Tongyeong coast from the 8th of June to the 22nd of September, 2016.

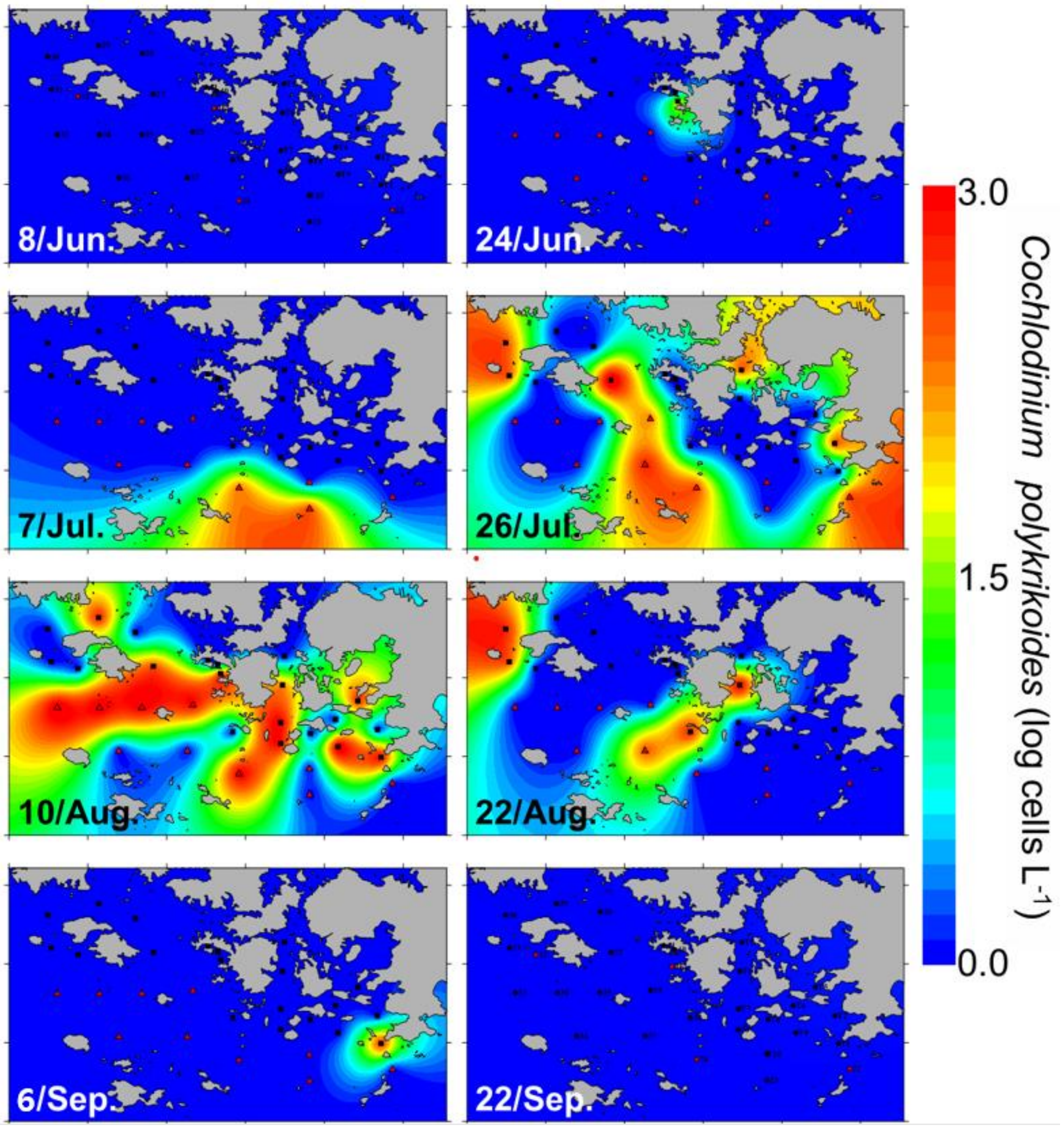

Figure S5. The 10-day accumulated precipitation before each field survey (A: Tongyeong, B: Namhae, and C: Goheung coasts). These data were obtained from the Korea Meteorological Administration. Light gray background indicates the time, when *C. polykrikoides* blooms were terminated in southern Korean coastal waters (KCW) due to typhoon events.

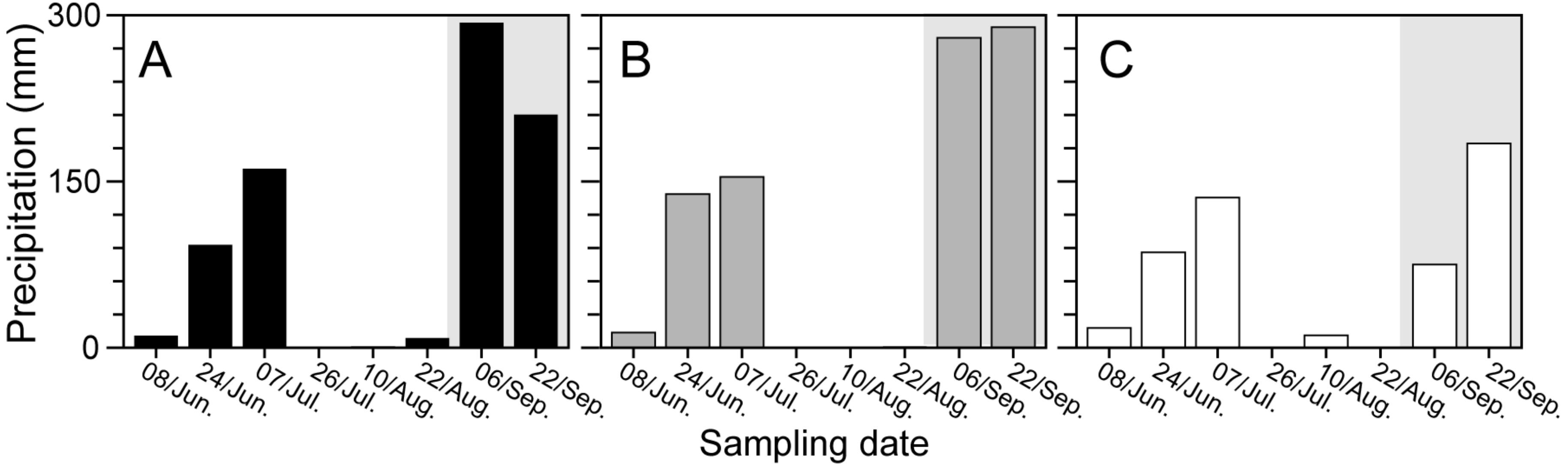

Figure S6. Spatial profiles (surface and bottom layers) of the environmental factors in the Goheung-Namhaedo area on the 7th of September. A: water temperature, B: salinity, C: chlorophyll-*a*, D: nitrite+nitrate; E: ammonium, F: phosphate, and G: silicate.

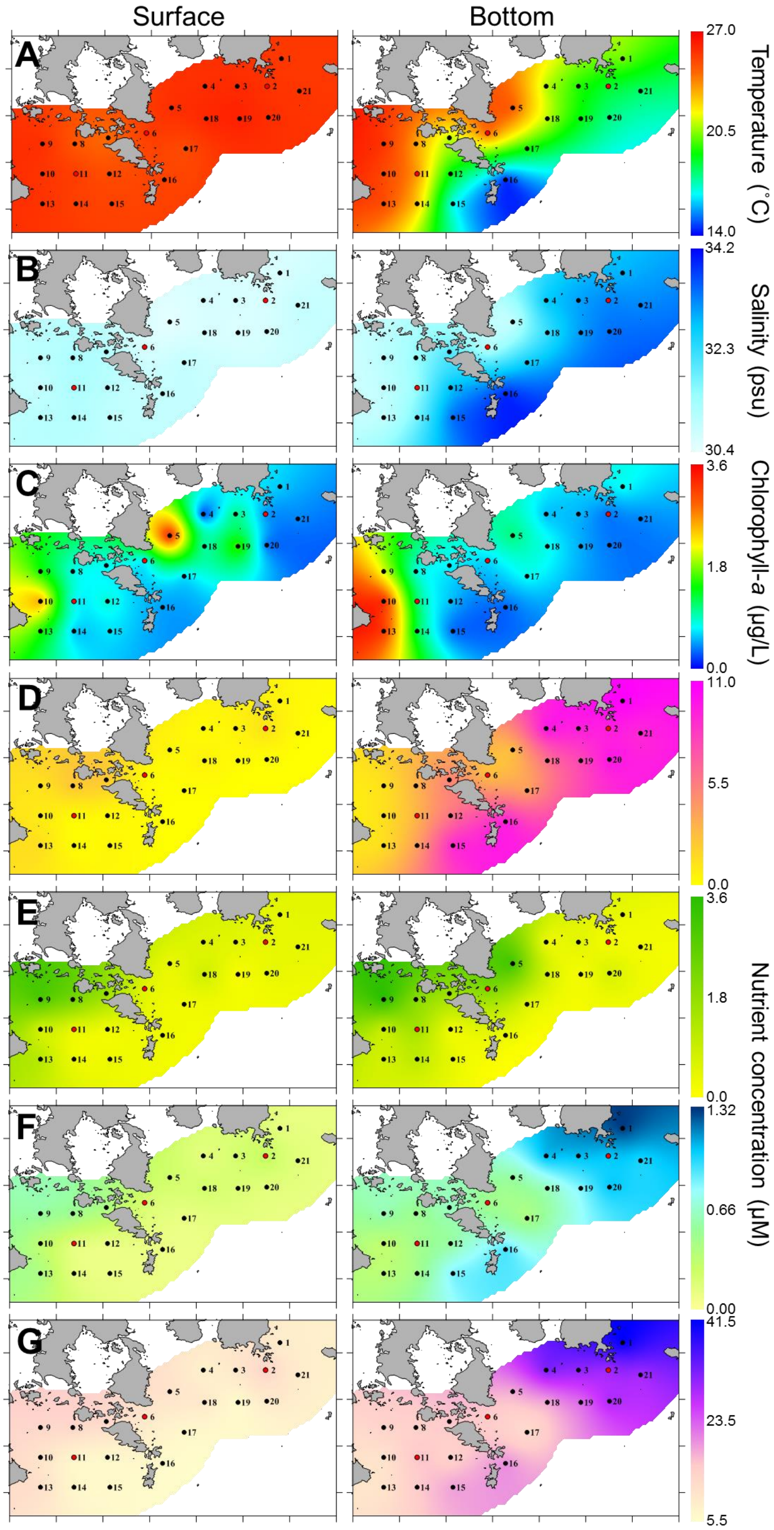

Supplement: Supplementary file 1 [file toxins-12-00390-s001.pdf]
